# The Value of Adjuvant Radiotherapy in the Treatment of Melanoma: A Multi-Center Analysis

**DOI:** 10.3390/cancers18182901

**Published:** 2026-09-08

**Authors:** Magdalena Fischer, Sabine Gerum, Falk Roeder, Matthias Brandlmaier, Laura Krecu, Julia Maria Ressler, Christoph Hoeller, Lukas Koch, Marie-Therese Dernoscheg, Marina Wanner, Johannes Lanbach, Van Anh Nguyen, Florentia Dimitriou, Reinhard Dummer, Alexander Egger, Johann W. Bauer, Katharina Schumann, Christian Posch, Peter Koelblinger

**Affiliations:** 1 Department of Dermatology and Allergology, Paracelsus Medical University, 5020 Salzburg, Austriam.brandlmaier@salk.at (M.B.);; 2 Department of Radiotherapy and Radiation Oncology, Paracelsus Medical University, 5020 Salzburg, Austriaf.roeder@salk.at (F.R.); 3 Institute of Research and Development of Advanced Radiation Technologies (radART), Paracelsus Medical University, 5020 Salzburg, Austria; 4 Department of Dermatology, Medical University Vienna, 1090 Vienna, Austria; 5 Department of Dermatology, Medical University Graz, 8010 Graz, Austria; 6 Department of Dermatology, Venerology and Allergology, Medical University Innsbruck, 6020 Innsbruck, Austria; 7 Department of Radiotherapy and Radiation Oncology, Medical University Innsbruck, 6020 Innsbruck, Austria; 8 Department of Dermatology, Faculty of Medicine, University Hospital of Zurich, 8091 Zurich, Switzerland; 9 Department of Surgical Oncology, The University of Texas MD Anderson Cancer Center, Houston, TX 77030, USA; 10 Department of Dermatology, Kantonsspital Aarau, 5001 Aarau, Switzerland; 11 Department of Dermatology and Allergy, School of Medicine, German Cancer Consortium (DKTK), Technical University of Munich, 81675 Munich, Germany; 12 Faculty of Medicine, Sigmund Freud University Vienna, 1020 Vienna, Austria; 13 Department for Dermatology, Clinic Hietzing, Vienna Healthcare Group, 1010 Vienna, Austria

**Keywords:** melanoma, radiotherapy, adjuvant therapy, immunotherapy

## Abstract

**Background:** Adjuvant radiotherapy (RT) may improve in-field regional tumor control in melanoma following resection of lymph node macrometastases. However, no significant effect on recurrence-free survival or overall survival has been demonstrated. The approval of adjuvant systemic immune checkpoint inhibitors and targeted therapies has significantly impacted the role of adjuvant RT. Its use in clinical practice has decreased, with heterogeneous treatment indications and approaches observed across institutions and regions. **Objectives:** The aim of the present study was to characterize the population of melanoma patients still undergoing adjuvant RT in the era of modern adjuvant systemic treatments, and to outline potential clinical scenarios in which it may continue to play a role in the future. **Patients and methods:** Fifty-eight patients treated with adjuvant RT were identified in five participating Austrian and Swiss dermato-oncologic centers. Treatment timing, toxicity, recurrence patterns, and survival outcomes were analyzed, and compared to 88 stage-matched, non-irradiated patients from a German cohort of patients who met formal RT criteria. **Results:** The majority of irradiated patients (53.5%) received RT before systemic therapy. The concurrent administration of adjuvant systemic therapy and radiotherapy did not increase the frequency of RT-associated toxicities (77.8% vs. 74.2%, *p* = 0.456). No significant differences in recurrence-free survival or overall survival were observed between irradiated and non-irradiated patients (*p* = 0.725 and *p* = 0.986, respectively). **Conclusions:** Although still recommended in certain melanoma guidelines, adjuvant RT is infrequently performed in the era of modern systemic treatments. Due to its exclusively locoregional efficacy, the application of adjuvant RT should be limited to clearly defined scenarios where systemic treatment options are limited and locoregional control is most important. The concurrent administration of adjuvant RT and systemic therapy is safe and should be preferred if a combined treatment strategy is chosen.

## Simple Summary

This study was undertaken to evaluate the current role of adjuvant radiotherapy after surgery for macrometastatic melanoma and to identify clinical situations in which it may still provide benefit. We found that adjuvant radiotherapy is now used infrequently but can be safely combined with modern systemic therapies without increasing radiotherapy-related toxicity. The findings suggest that routine use of adjuvant radiotherapy should be reserved for selected patients, particularly when systemic treatment options are limited, such as after progression during therapy or when there is no pathological response to neoadjuvant checkpoint inhibitor treatment, especially in patients with BRAF wild-type tumors.

## 1. Introduction

Optimal therapy of locoregionally metastasized melanoma may require a multimodal approach including surgical, systemic and radio-oncological treatment strategies. Adjuvant radiotherapy has long been considered an essential component of melanoma treatment, aiming to improve local control and survival. Studies performed before the era of effective systemic immune and kinase-directed treatment options demonstrated that adjuvant radiotherapy (RT) may reduce the risk of local in-field recurrence, however a positive impact on overall patient survival has not been demonstrated [1]. Potential synergistic effects of combined radio- and systemic therapy have been proposed in melanoma and other malignancies, suggesting that radiotherapy may enhance the immunogenicity of tumors, making them more susceptible to immune checkpoint inhibition [2,3,4,5,6,7]. Larger-scale controlled studies to confirm these systemic effects of radiotherapy, however, are lacking.

Since the approval of modern melanoma treatments—namely targeted therapy and checkpoint inhibitor-based immunotherapy—the role of adjuvant RT has changed [8,9,10]. While modern therapies were able to significantly improve recurrence-free survival in pivotal randomized trials [8,11], prospective studies addressing whether additional adjuvant RT may enhance local control or further improve survival outcomes have not been performed. Also, it is unclear in which specific clinical scenarios adjuvant RT is still routinely conducted and which RT protocols are typically deployed. Nevertheless, the recommendation for adjuvant radiotherapy is still included in certain melanoma guidelines. In particular, adjuvant RT is advocated in the presence of perinodal spread, large lymph node deposits, a high number of affected lymph nodes or lymphogenic recurrence [12].

The aim of this retrospective multi-center study was to re-define the value of adjuvant radiotherapy—in terms of application frequency and timing—in the era of modern melanoma therapy. In order to evaluate a potential impact of adjuvant RT on survival or recurrence, we compared patients undergoing adjuvant RT with stage-matched non-irradiated patients also meeting formal RT criteria outlined in the German melanoma guideline [13]. Concluding, currently still applicable adjuvant RT recommendations in this guideline on adjuvant radiotherapy were analyzed for their clinical value and ongoing significance in clinical practice.

## 2. Materials and Methods

### 2.1. Study Population

After approval by the local ethics committee (ethics committee number: 1207/2021 version 2, Salzburg), melanoma databases in five centers in Austria and Switzerland (hereafter also referred to as the “AUT” cohort) were queried for patients who received adjuvant RT between 2018 and 2021. Patient and tumor characteristics as well as details of RT regimens were retrospectively collected from the institutions’ electronic databases. The investigated data comprised patient demographics, details of the primary melanoma (type of melanoma, Breslow Index, localization, mutational and ulceration status) and details on the nodal basin (presence of micrometastases (<1 mm) and macrometastases (≥1 mm) upon pathological assessment, number of affected lymph nodes, site of node field, perinodal spread, largest diameter of metastasis and lymphogenic recurrence). Furthermore, it was assessed whether surgical procedures, such as sentinel lymph node biopsy, complete lymph node dissection (CLND) or selective excision of macrometastases, were performed previously. In all cases, radiotherapy was performed after prior complete R0 resection. Radiotherapy protocols were screened for the following information: dates, irradiated region, anatomical site, applied radiation technique (VMAT/IMRT/TOMO, 3-D CRT, electrons, 2D mega voltage or 2D ortho voltage), the number of fractions, the single dose per fraction and the total radiation doses. Normo-fractionated (1.8–2 Gy) and hypo-fractionated (>2–4 Gy) radiation protocols were distinguished. Only patients irradiated in the lymph node basin were included in the analyses, patients who received irradiation only at the site of primary melanoma were excluded. Data were analyzed as to whether RT was performed before, after, or simultaneously with systemic therapy (ST). From the data derived, three groups were generated: the first group included patients who were irradiated before adjuvant systemic treatment (upfront RT), the second one consisted of patients receiving a systemic therapy in parallel with RT (concurrent RT), and patients in the third group received ST before RT (late RT cohort). All patients in the late cohort were irradiated due to a recurrence during or after having received systemic therapy.

Any adverse events related to irradiation and interruptions of four or more days in the radiation treatment caused by side effects were documented.

Additionally, data of the AUT cohort were compared with stage-matched non-irradiated melanoma patients who received adjuvant systemic therapy with PD1-antibodies or BRAF+MEK inhibitors. These data were retrieved from a large multi-center cohort, subsequently referred to as the “TUM” (Technical University Munich) dataset [13]. RT patients in the AUT cohort who received adjuvant RT due to recurrence after systemic treatment (“late RT” subgroup) were excluded in these analyses to ensure comparability. Due to the multi-center characteristic of the TUM study including Austrian, German and Swiss centers, duplicate data (216 patients) in the TUM dataset had to be removed. Thereafter, 982 melanoma patients remained in the TUM cohort, of which 172 (17.5%) had undergone RT and were therefore also excluded. Of the remaining 810 non-irradiated patients, we identified a sub-cohort of patients who met formal criteria for adjuvant radiotherapy as outlined in the German melanoma guideline [12]. These are as follows: pericapsular lymph node growth, three or more lymph nodes affected or lymphogenic recurrence. Particularly large lymph node metastases (three cm or larger) are also considered an indication for adjuvant radiotherapy [12]. Due to lack of data regarding the exact size of lymph node metastases and the presence or absence of lymphogenic recurrence in the control group, we only included patients featuring either pericapsular growth and/or three or more lymph node metastases. Eventually, 88 TUM patients with lymph node metastases fulfilling at least one of the above-mentioned RT criteria were included.

Stage of disease and side effects were graded according to the AJCC 8th edition and the Common Terminology Criteria for Adverse Events (CTCAE) version 5.0, respectively [14]. The date of recurrence was defined as the date of histologic (biopsy) or radiographic examination. Distant and locoregional recurrence were distinguished, and the latter was sub-classified into regional recurrence (recurrence in the lymph node basin/draining lymph ducts) and local, i.e., affecting the field of the primary melanoma.

### 2.2. Statistical Analysis

Descriptive statistics were used to outline patient and tumor characteristics.

Two primary endpoints were defined: recurrence-free survival (RFS) and overall survival (OS). Patients in whom the defined endpoint (relapse or death) did not occur within the defined period were censored at the time of the last follow-up examination.

Within the AUT group, RFS was calculated for both the upfront and concurrent RT subgroup. The log-rank (Mantel–Cox) test was used for survival comparisons and the results were visualized with Kaplan–Meier curves. For the comparison between upfront and concurrent radiotherapy, the respective start date of the first adjuvant therapy, either primary adjuvant radiotherapy or primary adjuvant systemic therapy, whichever came first, was used for survival calculations.

Regarding patients in the late RT cohort, RFS 2 was calculated from initiation of adjuvant RT after first relapse to the time of subsequent relapse.

To assess possible differences between irradiated and non-irradiated risk populations, we compared the RFS and OS distributions of the AUT and TUM group by log-rank testing. RFS and OS comparisons are exploratory and unadjusted.

All statistical tests were two-sided with a significance level at 0.05. Data processing was performed with Excel 2016 for Microsoft Windows^®^ (Microsoft Corporation, Redmond, WA, USA). For all statistical calculations, GraphPad Prism version 10.0.0 for Windows, GraphPad Software, Boston, MA, USA (www.graphpad.com) was used.

## 3. Results

### 3.1. Results Dataset AUT

#### Patient Population

A total of 58 patients who received adjuvant RT were identified (18 (31%) female, 40 (69%) male) with a mean age of 65 years (range 27–88 years) at the time of irradiation. Baseline characteristics are summarized in Table 1. Median follow-up was 26 months (range 3–58 months).

Of the primary melanomas, 39 (67.2%) were cutaneous, eight (13.8%) were acral, and one (1.7%) was mucosal. For 10 (17.2%) patients, no information on the primary tumor was provided. Adjuvant systemic treatment regimens included nivolumab in 35 patients (60.3%), pembrolizumab in seven (12.1%) and BRAF/MEK inhibitors in 16 (27.6%). Adjuvant systemic therapy was administered for a median of six months.

The median size of the largest lymph node deposit was 19 mm. Nodal deposits greater than or equal to three cm in diameter were detected in 19 (32.8%) patients. Sentinel lymph node biopsy and CLND were performed in 28 (48.3%) and 41 (70.7%) patients, respectively.

### 3.2. Radiotherapy

Regarding the indication for adjuvant RT, in 33 patients (56.9%), lymph node metastases with trans-capsular growth were present, in 21 (36.2%) three or more lymph nodes were affected, and 10 patients (17.2%) were irradiated due to lymphogenic recurrence (see Table 1). Macrometastases were detected in 43 patients (74.1%), of which 79% underwent therapeutic lymph node dissection, whereas in 25.6% a selective excision of isolated macrometastasis was performed before irradiation.

**Figure 1:**
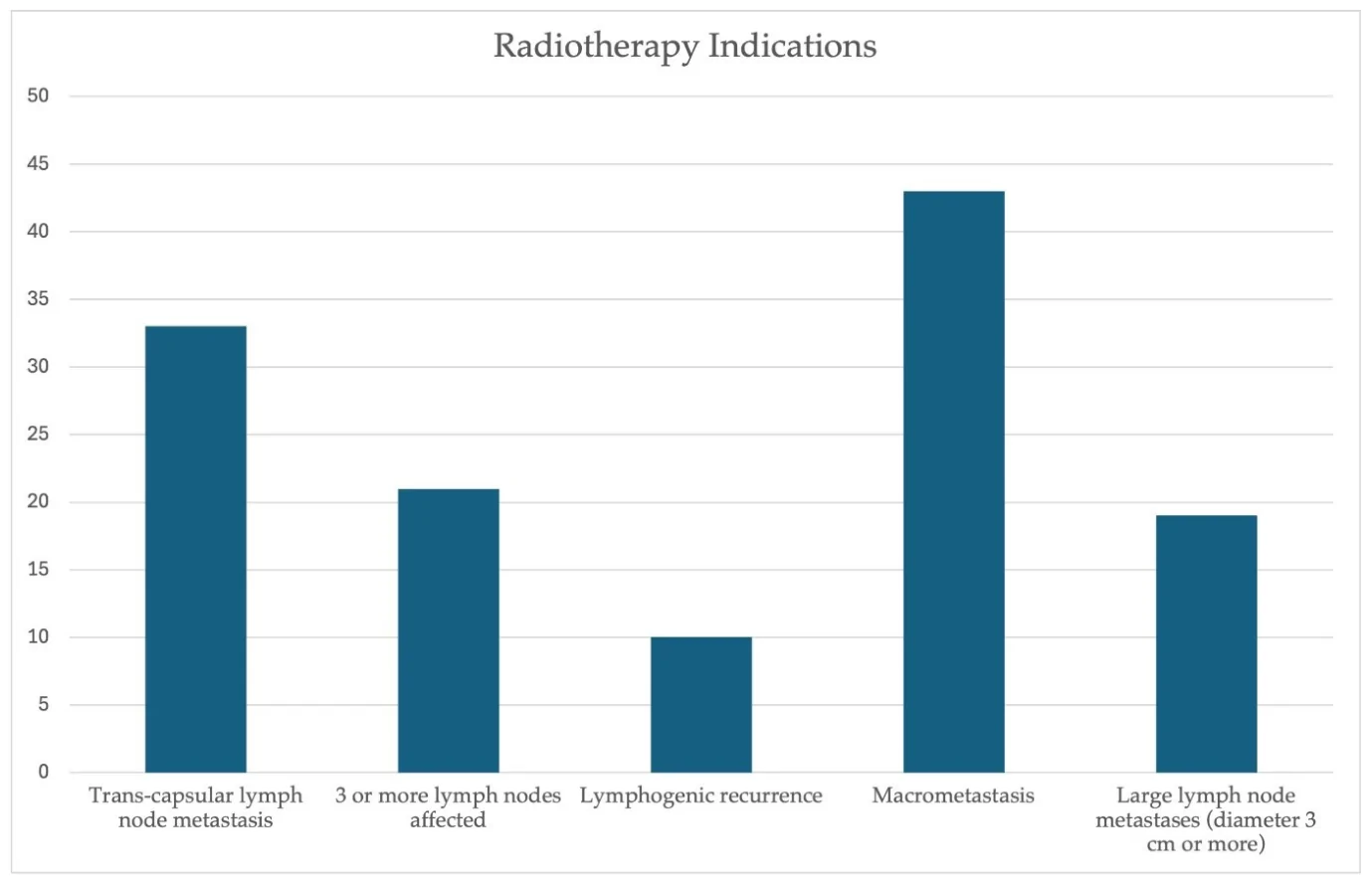
RT indications (absolute numbers).

As illustrated in Figure 1, in several patients multiple RT indications were present.

Irradiation was performed according to a hypo-fractionated protocol in 41 (70.7%) and to a normo-fractionated protocol in 17 patients (29.3%). Comparative RFS analyses did not show a statistical difference with regard to type of fractionation (*p* = 0.6763). Radiation details are depicted in Table 2.

Forty-five patients (77.6%) experienced RT-associated acute toxicity (Table 3), with grade one or two radiodermatitis being most common. Grade three or four toxicities occurred in two patients (3.5%). Thirteen patients developed two or more RT-associated toxicities, and one patient experienced persistent lymphedema, which was classified as a grade III late toxicity. In one patient, radiotherapy was interrupted for a period longer than four days (Table 3). In the concurrent RT group, 13 patients received nivolumab, two pembrolizumab and three BRAF+MEK inhibitors.

RT was performed prior to systemic therapy in 53.5% of patients. With a median interval of 17.5 days, most of these patients received systemic adjuvant therapy shortly after completion of adjuvant RT. The percentage of patients who received RT concurrent with or after completion of systemic therapy were 31 and 15.5%, respectively. A comparison of risk profiles of patients with upfront and concurrent RT is depicted in Supplementary Table S1.

Eighteen (58.1%) and ten (55.6%) patients with upfront and concurrent RT experienced disease recurrence after adjuvant RT, respectively, most often affecting lymph nodes or soft tissue (Table 4). Median time to recurrence was 22.5 months. Among the progressive cases, seventeen had locoregional metastases (lymph node or field of primary melanoma), eight demonstrated a combination of locoregional and distant progression, and eleven experienced distant progression only. Nineteen patients recurred with lymph node metastasis (regional or distant), whereas four showed a local recurrence within the field of the primary melanoma. Distant metastases most frequently involved the skin, soft tissue, muscle, and/or non-regional lymph nodes.

**Figure 2:**
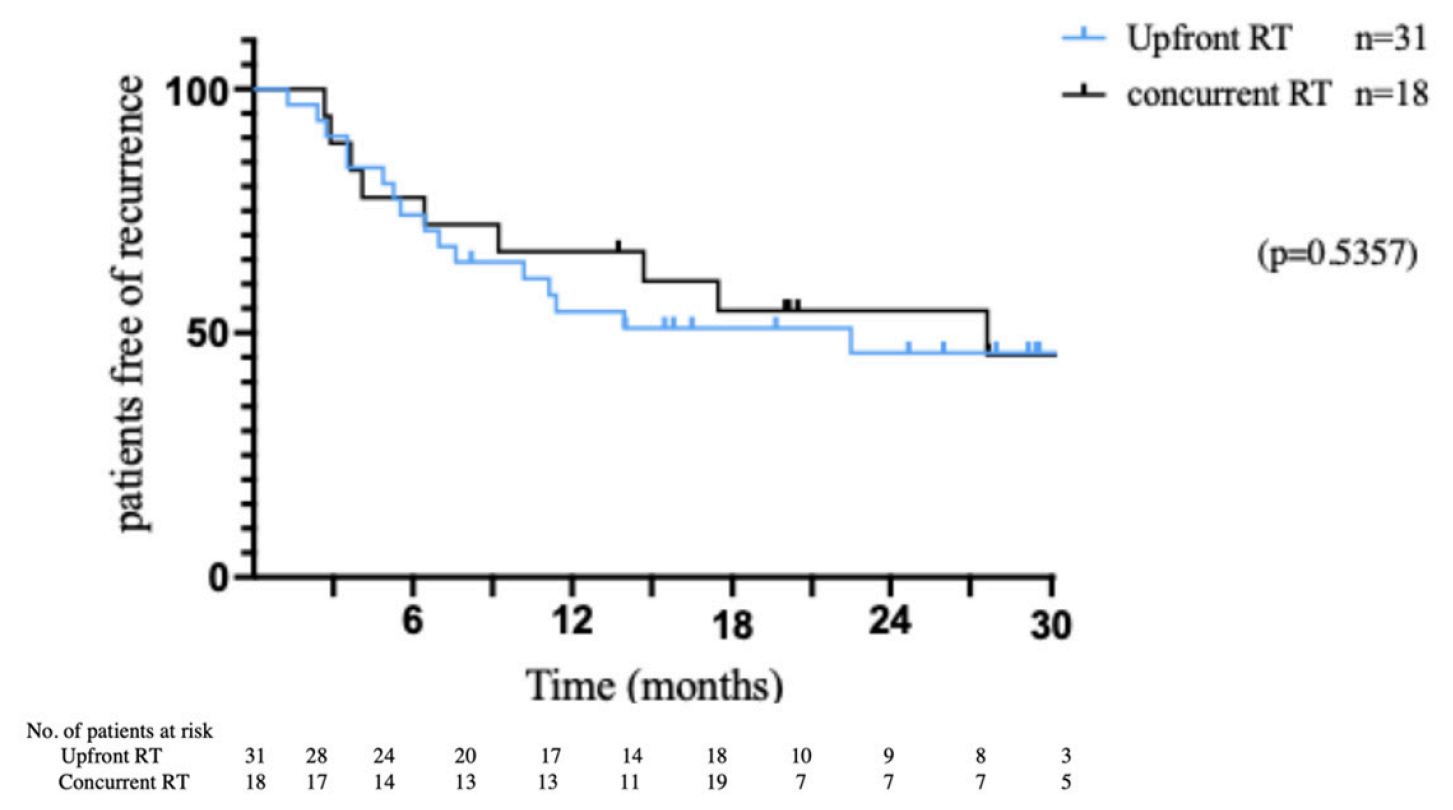
Comparison of RFS (upfront RT vs. concurrent RT).

RFS rates in upfront RT patients did not differ significantly compared to patients who were irradiated in parallel with systemic therapy (*p* = 0.5357, Figure 2). Recurrence-free survival for concurrent/upfront RT was 66.7/58.4% and 38.9/32.3% at 1 and 2 years after initiation of adjuvant therapy, respectively.

Also, frequency of acute radiotherapy-associated toxicities was similar in the concurrent and upfront RT cohort (77.8 vs. 74.2%, respectively (*p* = 0.4559), Table 3).

In nine patients (15.5%), adjuvant radiation was conducted after systemic therapy. The indication for adjuvant RT was disease recurrence during or after systemic therapy in all of these patients. In this late RT group, the average time between relapse and the initiation of adjuvant RT was 7.5 months. Four patients (44.4%) relapsed again despite adjuvant RT; two with locoregional metastases and two with distant metastases. The RFS2 rates were 88.9%, 88.9% and 74.1% after 12, 24 and 36 months, respectively.

#### Comparison of AUT and TUM Cohorts

A total of 49 patients who received upfront or concurrent adjuvant radiotherapy in the AUT cohort were compared to 88 non-irradiated patients from the TUM cohort meeting formal RT criteria as outlined earlier. Patient and tumor characteristics of the TUM cohort are summarized in Table 5.

**Figure 3:**
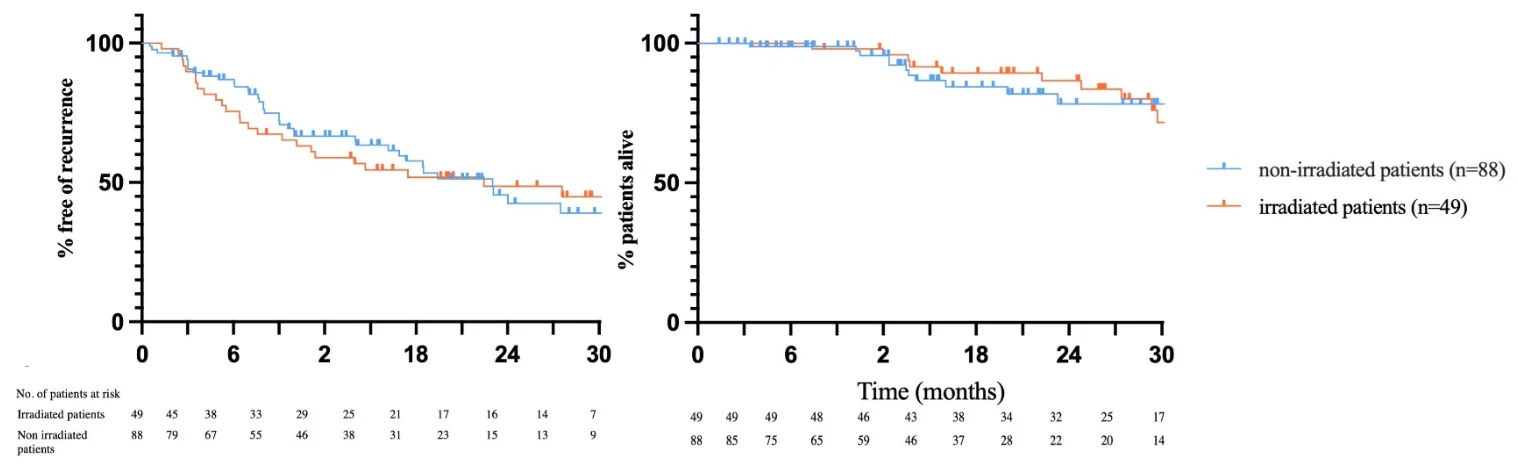
Comparison of RFS (**left**) and OS (**right**) of irradiated patients (primary and concurrent RT) vs. non-irradiated patients with formal RT indication (TUM cohort); (*p* = 0.7249 and *p* = 0.9862, respectively).

A comparison of recurrence-free survival yielded similar outcomes for irradiated and non-irradiated patients (*p* = 0.7249, 95% CI 0.562–1.496). Median RFS was 23 and 22.5 months in the non-irradiated and irradiated group, respectively (Figure 3 left). OS was similar in irradiated and non-irradiated patients (*p* = 0.9862; Figure 3 right, 95% CI 0.48–2.4).

A total of 24 non-irradiated patients (27.3%) meeting formal RT criteria (TUM group) and 41 irradiated patients (70.7%) in the AUT cohort underwent CLND. In dissected patients, recurrence did not occur significantly more frequently in non-irradiated patients compared to irradiated patients (10/24 patients (41.7%) vs. 25/41 patients (61%), respectively; *p* = 0.1886). Among the patients who received CLND, an average of 3.4 and 3.6 lymph nodes were affected in the RT and the TUM subgroup, respectively.

## 4. Discussion

Adjuvant systemic treatment with immune checkpoint or kinase inhibitors has been considered the standard of care in the management of completely resected stage III and IV melanoma since approval of nivolumab, pembrolizumab and dabrafenib/trametinib in 2019 [8,11]. Due to the significant improvement of RFS associated with novel systemic therapies, the role of adjuvant RT has certainly changed [15,16,17]. Thorough patient selection and risk stratification are necessary to identify individuals who may still benefit from adjuvant radiotherapy despite the availability of highly efficacious systemic treatments. To determine which subgroups of patients still receive adjuvant RT alongside modern systemic treatment, we analyzed the current clinical practice. Also, we investigated the impact of adjuvant RT on survival outcomes through comparison with stage-matched patients who received systemic adjuvant treatment but no adjuvant RT.

Although various recently published international melanoma guidelines (NCCN, EADO/EDF/EORTC, ESMO) no longer recommend adjuvant RT [18,19,20], it is still recommended in the current German (AWMF) melanoma guideline with the second highest level of recommendation for patients who have undergone CLND and feature at least one high-risk criterion for locoregional recurrence as outlined earlier [12]. These recommendations have remained unchanged despite the availability and routine use of modern adjuvant systemic therapies. In accordance with the AWMF guideline recommendations, we found that adjuvant RT is still most frequently conducted when trans-capsular growth or three or more affected lymph nodes have been detected histologically. Both hypo- and normo-fractionated radiation protocols were routinely applied in the adjuvant setting, which is in line with findings by Holtkamp et al. who reported no difference between regarding nodal field control rates in stage III melanoma patients [21].

In contrast to radiotherapy, adjuvant systemic therapies are associated with a significant RFS benefit. Hence, they are also recommended (with the highest level of recommendation) in the various melanoma guidelines. However, despite the current recommendation for adjuvant RT, the AWMF guideline does not address at all at which timepoint adjuvant RT should be conducted in the context of adjuvant systemic treatment [12]. In our patient cohort, upfront adjuvant RT was more frequently conducted than concurrent radio- and systemic therapy. However, as we did not observe an increased rate of RT-related toxicities in patients who underwent concurrent treatment, we propose not to delay adjuvant systemic treatment in those high-risk cases in which additional RT is considered to improve local control besides systemic adjuvant treatment.

Regional nodal recurrence is common in patients at high risk of relapse, fulfilling formal RT criteria outlined above [22,23]. Although adjuvant RT has been demonstrated to mitigate the risk of early nodal recurrence without an impact on RFS and OS, regional recurrence occurred in 13 of 49 patients (26.5%) who underwent upfront or concurrent adjuvant RT in our cohort. In the pivotal adjuvant RT trial by Burmeister et al., a comparable lymph-node field relapse rate of 18.3% (20/109 patients) was reported [24]. As presumed, we did not observe any improvement in either RFS or OS through the addition of adjuvant RT, confirming data in the pre-systemic therapy era.

We identified three common treatment scenarios in which adjuvant RT was conducted. First, the majority of patients (53%) received upfront RT followed by immune checkpoint inhibitor treatment. Second, 31% of patients received radiation concurrent with systemic therapy. The remaining 16% of patients were those who received adjuvant RT subsequent to surgery of locoregional melanoma recurrence diagnosed during or after termination of adjuvant systemic treatment. Regarding the latter subgroup, a recent retrospective multi-center analysis by Bhave et al. [25] showed that adjuvant RT can improve locoregional disease control (lr-RFS2) in patients who progressed during or after adjuvant PD-1 inhibition, while no effect on distant metastasis-free survival was detected. In our small subgroup of late RT patients (n = 9), only two locoregional recurrences occurred, similar to the results reported by Bhave and colleagues (two locoregional second recurrence events in 24 irradiated patients) [25]. These findings imply a continuing role for adjuvant RT in clinical scenarios, in which locoregional disease control becomes the primary goal of treatment after adjuvant systemic therapy has failed. Such a scenario can also occur in the course of neoadjuvant treatment approaches, which were recently investigated in pivotal trials such as SWOG 1801 and NADINA and have fundamentally changed the management of patients with melanoma macrometastases [26,27]. Both in the NADINA and the precedent PRADO trial [28], adjuvant radiotherapy was possible according to patient and physician decisions in patients not reaching a major pathological response after pre-operative combined checkpoint inhibition. Adjuvant RT was actually conducted in 8 out of 21 patients with a pathological non-response (pNR) in the PRADO trial [28]. Although this subgroup of patients has not yet been reported on separately, adjuvant RT may be particularly useful for local disease control in this scenario, especially in patients with BRAF wild-type tumors for whom no alternative systemic adjuvant treatment is available.

The main limitation of our study is inherently its retrospective design itself. Moreover, the radiotherapy cohort was relatively small despite multi-center patient inclusion, which somehow confirms that the utilization of adjuvant RT is increasingly limited to specific clinical scenarios.

## 5. Conclusions

We observed that adjuvant radiotherapy is still conducted in selected patients, mostly in accordance with guideline recommendations, and, to our surprise, more often as an upfront treatment strategy. We found that RT-associated side effects are common but mild in the majority of cases. Similar to previous reports, we did not detect a benefit regarding RFS or OS in irradiated patients. We therefore recommend that if a decision for first-line adjuvant RT is made, i.e., in high-risk constellations where local tumor-control is of utmost importance, a concurrent approach combining adjuvant radio- and systemic therapy should be selected. Upfront RT instead of systemic therapy may be obsolete unless there are absolute contraindications to systemic therapy.

Future guideline recommendations—particularly in the forthcoming revised version of the German Melanoma Guideline, which serves as a key reference for clinical practice in Austria and Germany—should limit the routine use of adjuvant RT to clearly defined scenarios where systemic treatment options are limited. These include cases of disease progression following adjuvant therapy or a pathological non-response after neoadjuvant checkpoint inhibitor treatment, especially in patients with BRAF-negative tumors [29]. Prospective studies are needed to appropriately confirm the efficacy of radiotherapy in these particular situations.

## Figures and Tables

**Table 1:** Patient and tumor characteristics (AUT cohort).

Patients Receiving Adjuvant Radiotherapy (n = 58)	
Localization of primary tumor	n (% of total)
Upper extremity	1 (1.7)
Lower extremity	23 (39.7)
Trunk	15 (25.9)
Head	9 (15.5)
Unknown Primary	10 (17.2)
Age	years
mean	65
Breslow Index	mm
Median	2.6
Mean	3.5
Mutational status	n (% of total)
BRAF	32 (55.2)
NRAS	16 (27.6)
cKIT	0
Wild-type	10 (17.2)
Diameter of largest lymph node metastasis	mm
Median	19
Mean	24.9
Diameter of largest lymph node metastasis	n (% of total)
<3 cm	27 (46.6)
≥3 cm	19 (32.8)
n.a.	12 (20.7)
Prior surgical treatment	n (% of total)
Sentinel lymph node biopsy	28 (48.3)
Complete lymph node dissection	41 (70.7)
Number of affected lymph nodes	n (% of total)
0	1 (1.7)
1–2	34 (58.6)
3–4	8 (13.8)
5 or more	13 (22.4)
n.a.	2 (3.5)
Pericapsular lymph node growth	n (% of total)
Yes	33 (56.9)
n.a.	2 (3.5)
Presence of macrometastases	n (% of total)
Yes	43 (74.1)
Lymphogenic recurrence before RT	n (% of total)
	10 (17.2)
AJCC stage at start of RT	n (% of total)
IIIB	16 (27.6)
IIIC	30 (51.7)
IIID	3 (5.2)
IV	8 (13.8)
n. a.	1 (1.7)

**Table 2:** Sites of radiation, radiation techniques and radiation doses.

Site of Radiation	Number of Patients (n Total = 58) n; Percentage of Total
Primary tumor only	0
Regional lymph node basin	56 (96.6%)
Primary tumor and regional lymph node basin	2 (3.5%)
**Radiation Technique**	
VMAT/IMRT/TOMO	21 (36.2%)
3-D CRT	23 (39.74%)
Electrons	1 (1.7%)
2D Mega (High) Voltage	11 (19%)
2D Ortho Voltage	1 (1.7%)
Unknown	1 (1.7%)
**Radiation Dose**	Gy
Single	
Mean/median (range)	3.7/2.4 (1.8–4)
Cumulative	
Mean/median (range)	48.4/48 (35–70)
Number of fractions	n
Mean/median (range)	20.7/20 (10–35)

**Table 3:** Toxicities associated with RT. Multiple responses possible, as some patients had several toxicities. Total number of irradiated patients = 58.

Patients with Any Acute Toxicity	45 (77.6%)	Upfront RT (n = 31)	Concurrent RT (n = 18)	Late RT (n = 9)
	Number of Patients (n Total = 58) n			
Radiodermatitis	39 (I° n = 25, II° n = 14, III° n = 0)	21 (I° n = 14; II°7)	14 (I° n = 7, II° n = 7)	4 (I° n = 4)
Abscess	1 (IV°)	0	1 (IV°)	0
Mucositis	3 (I° n = 1, II° n = 2)	2 (II° n = 2)	1 (I° n = 1)	
Dysgeusia	3 (I° n = 2, III° n = 1)	2 (I° n = 1; III° n = 1)	1 (I° n = 1)	
Xerostomia	1 (I°)	1 (I°)	0	
Fatigue	2 (I°)	0	2 (I°)	0
Lymphedema	5 (I° n = 1, II° n = 3, III° n = 1)	5 (I° n = 1, II° n = 3, III° n = 1)	0	0
Any late toxicity > III°	1	1	0	0
Lymphedema III°	1	1	0	0
Patients with 2 or more toxicities	13	9	4	0
Patients with RT interruption for 4 or more days due to RT-toxicities	1	1	0	0

**Table 4:** Areas of recurrence in patients who received upfront or concurrent RT (total number of patients with upfront/concurrent RT = 49).

Area of First Recurrence (Multiple Areas Possible)	Number of Patients with Recurrence (n Total = 28) n (%)
Locoregional metastases	17 (60.7)
Regional (lymphatic system)	13 (46.4)
Local (site of primary melanoma)	4 (14.3)
Distant metastases	
Soft tissue/distant lymph nodes	6 (21.4)
Distant organs	6 (21.4)
Lung	7 (25)
CNS	3 (10.7)

**Table 5:** Patient and tumor characteristics of non-irritated patients meeting formal RT criteria (TUM cohort).

Non-Irradiated Patients with Formal RT Criteria (n = 88)	
**Age**	Years
Median	60
Mean	59.7
**Breslow Index**	mm
Median	2.9
Mean	4.7
**Mutational Status**	n (% of total)
BRAF	32 (36.4)
**Diameter of largest lymph node metastasis**	mm
Median	19
Mean	24.9
**Prior surgical treatment**	n (% of total)
Complete lymph node dissection	24 (27.3)
**Number of affected lymph nodes**	n (% of total)
0	0 (0)
1–2	27 (30.7)
3–4	43 (48.9)
5 or more	18 (20.45)
n.a.	0 (0)
**Pericapsular lymph node growth**	n (% of total)
Yes	47 (53.4)
**AJCC stage at start of IT**	n (% of total)
IIIA	4 (4.6)
IIIB	14 (15.9)
IIIC	62 (70.45)
IIID	4 (4.6)
IV	4 (4.6)
**Type of systemic therapy**	n (% of total)
Nivolumab	43
Pembrolizumab	30
BRAF+MEK inhibitors	15
**Follow-up**	Time (months)
Mean	26.6
Median	23.4

## Data Availability

The data presented in this study are available from the corresponding author upon reasonable request.

## Supplementary Materials

The following supporting information can be downloaded at https://www.mdpi.com/article/10.3390/cancers18182901/s1. Supplementary Table S1. comparison of risk profiles of patients with upfront and concurrent RT.

## Abbreviations

The following abbreviations are used in this manuscript:

AUT cohort	five centers in Austria and Switzerland
AWMF	Arbeitsgemeinschaft der Wissenschaftlichen Medizinischen Fachgesellschaften
CLND	complete lymph node dissection
CTCAE	Common Terminology Criteria for Adverse Events
EADO	EADO
EDF	European Dermatology Forum
ESMO	European Society for Medical Oncology
EORTC	European Organisation for Research and Treatment of Cancer
Gy	Gray
IMRT	intensity-modulated radiation therapy
NCCN	National Comprehensive Cancer Network
RFS	recurrence-free survival
RT	radiotherapy
ST	systemic therapy
TOMO	tomotherapy
TUM	Technical University Munich
OS	overall survival
pNR	pathological non-response
VMAT	volumetric modulated arc therapy
