# Supplementary material for: The Value of Adjuvant Radiotherapy in the Treatment of Melanoma: A Multi-Center Analysis
Source: Cancers (Basel). 2026 Sep 8;18(18):2901. doi: 10.3390/cancers18182901 (PMC13604068; doi:10.3390/cancers18182901)
Supplement: Supplementary file 1 [file cancers-18-02901-s001.zip › cancers-4487906-supplementary.pdf]

	Upfront RT (n=31)	Concurrent RT (n=18)
Breslow Index	mm	mm
Median/mean	2.6/3.5	2.4/3.24
Diameter of largest lymph node metastasis	mm	mm
Median/mean	19/24.9	25/26.9
Presence of lymph node metastasis ≥ 3cm	%	%
	35.5	44.4
Patients with 3 or more affected lymph nodes	%	%
	25.8	50
Patients with pericapsular lymph node growth	%	%
	61.3	55.6
Presence of macrometastases	%	%
	83.9	66.7

Supplementary Table S1. comparison of risk profiles of patients with upfront and concurrent RT
